# Prediction of lymphovascular invasion in esophageal squamous cell carcinoma by computed tomography-based radiomics analysis: 2D or 3D ?

**DOI:** 10.1186/s40644-024-00786-5

**Published:** 2024-10-17

**Authors:** Yang Li, Xiaolong Gu, Li Yang, Xiangming Wang, Qi Wang, Xiaosheng Xu, Andu Zhang, Meng Yue, Mingbo Wang, Mengdi Cong, Jialiang Ren, Wei Ren, Gaofeng Shi

**Affiliations:** 1 Department of Computed Tomography and Magnetic Resonance Imaging, The Fourth Hospital of Hebei Medical University, Shijiazhuang, Hebei Province 050011 China; 2 Department of Radiotherapy, The Fourth Hospital of Hebei Medical University, Shijiazhuang, Hebei Province China; 3 Department of Pathology, The Fourth Hospital of Hebei Medical University, Shijiazhuang, Hebei Province China; 4 Department of Thoracic Surgery, The Fourth Hospital of Hebei Medical University, Shijiazhuang, Hebei Province China; 5 Department of Radiology, The Hebei Children’s Hospital, Shijiazhuang, Hebei Province China; 6 GE Healthcare China, Beijing, China

**Keywords:** Esophageal squamous cell carcinoma, Radiomics, Computed tomography, Lymphovascular invasion

## Abstract

**Background:**

To compare the performance between one-slice two-dimensional (2D) and whole-volume three-dimensional (3D) computed tomography (CT)-based radiomics models in the prediction of lymphovascular invasion (LVI) status in esophageal squamous cell carcinoma (ESCC).

**Methods:**

Two hundred twenty-four patients with ESCC (158 LVI-absent and 66 LVI-present) were enrolled in this retrospective study. The enrolled patients were randomly split into the training and testing sets with a 7:3 ratio. The 2D and 3D radiomics features were derived from the primary tumors’ 2D and 3D regions of interest (ROIs) using 1.0 mm thickness contrast-enhanced CT (CECT) images. The 2D and 3D radiomics features were screened using inter-/intra-class correlation coefficient (ICC) analysis, Wilcoxon rank-sum test, Spearman correlation test, and the least absolute shrinkage and selection operator, and the radiomics models were built by multivariate logistic stepwise regression. The performance of 2D and 3D radiomics models was assessed by the area under the receiver operating characteristic (ROC) curve. The actual clinical utility of the 2D and 3D radiomics models was evaluated by decision curve analysis (DCA).

**Results:**

There were 753 radiomics features from 2D ROIs and 1130 radiomics features from 3D ROIs, and finally, 7 features were retained to construct 2D and 3D radiomics models, respectively. ROC analysis revealed that in both the training and testing sets, the 3D radiomics model exhibited higher AUC values than the 2D radiomics model (0.930 versus 0.852 and 0.897 versus 0.851, respectively). The 3D radiomics model showed higher accuracy than the 2D radiomics model in the training and testing sets (0.899 versus 0.728 and 0.788 versus 0.758, respectively). In addition, the 3D radiomics model has higher specificity and positive predictive value, while the 2D radiomics model has higher sensitivity and negative predictive value. The DCA indicated that the 3D radiomics model provided higher actual clinical utility regarding overall net benefit than the 2D radiomics model.

**Conclusions:**

Both 2D and 3D radiomics features can be employed as potential biomarkers to predict the LVI in ESCC. The performance of the 3D radiomics model is better than that of the 2D radiomics model for the prediction of the LVI in ESCC.

**Supplementary Information:**

The online version contains supplementary material available at 10.1186/s40644-024-00786-5.

## Background

Esophageal carcinoma (EC) is the 7th leading cause of cancer morbidity and the 6th leading cause of cancer mortality globally [1]. Most reported cases are in Eastern Asia and Africa, with China alone accounting for an estimated 307,000 cases [1, 2]. Esophageal squamous cell carcinoma (ESCC) is East Asia’s most prevalent malignancy type. Since early ESCC has no specific symptoms, most tumors are discovered late in their progression, when treatment choices are limited and a cure is impossible. Endoscopic screening for ESCC in high-prevalence areas in China has significantly reduced deaths, and survival rates in China have been improved for decades [3, 4].

Lymphovascular invasion (LVI) is a histological characteristic linked to physiologically aggressive tumors, increasing the risk of local cancer micrometastasis. Even if lymph node metastases are absent, LVI remains a significant prognostic factor for patients with EC [5]. EC patients in the same period can be further classified into high-risk patients by LVI, which may allow for improved multimodality treatment [6]. It is preferable to use endoscopic resection (ER), such as endoscopic mucosal resection (EMR) and endoscopic submucosal dissection (ESD), for treating T1a-muscularis mucosae or T1b-submucosa (MM/SM1) stage ESCC [7]. Histopathological assessment of endoscopically resected specimens plays a crucial role in the decision-making process regarding the need for supplementary therapeutic interventions [8]. However, additional prophylactic treatment is necessary if LVI is found in the ER specimen [9]. If the pathological diagnosis after ER shows epithelium/lamina propria mucosa invasion or pathological T1a-muscularis mucosae invasion without LVI or droplet infiltration, these patients are proposed to be observed without further intervention. If the pathological diagnosis following ER shows pT1b-submucosal infiltration (with LVI or droplet infiltration), it indicates a significant probability of metastases, and additional treatment, including esophagectomy and chemoradiation therapy, is recommended [7]. It follows that preoperative identification of the LVI status can optimize the choice of treatment modality. As a noninvasive imaging technique, contrast-enhance CT (CECT) is the best imaging tool for showing the local invasion of surrounding structures, which helps decide surgical resection suitability [10]. However, LVI is only identified in pathologic specimens following surgical resection. Therefore, preoperative LVI prediction using conventional imaging techniques is a great challenge.

Radiomics refers to extracting and analyzing sophisticated quantitative features from medical images, allowing for high-throughput analysis [11]. In recent years, radiomics analysis has gained popularity as a noninvasive quantitative evaluation approach for esophageal tumors [12]. Previous studies have highlighted the potential of radiomics as a biomarker for predicting LVI in gastrointestinal cancers [13–18]. Tumors are represented with multiple layers on CT images, providing the option to outline the three-dimensional (3D) whole-volume tumor as the regions of interest (ROIs) or select a typical two-dimensional (2D) layer as the ROIs. Intuitively, the 3D mode provides the advantage of encompassing the entire tumor, while the 2D mode is more accessible to obtain, less labor-intensive, less complex, and faster to compute. When extracting radiomics features, a fundamental trade-off between the two outlining modes is necessary. In a previous study, we discovered a similar performance of 2D versus 3D radiomics models in identifying T1-2 versus T3-4 stage ESCC [20]. Meng et al. [16] showed that the 2D radiomics model performed slightly better than the 3D radiomics model in predicting the LVI of GC. In our previous study, we demonstrated the predictive capability of the 3D radiomics features derived from CECT images in determining the LVI status of ESCC [19]. However, the performance differences between 2D and 3D radiomics models in predicting LVI in ESCC have yet to be investigated.

Therefore, the primary objective of this study was to evaluate the potential value of the 2D radiomics model for predicting LVI in ESCC and to compare the performance differences between 2D and 3D radiomics models.

## Materials and methods

### Patients

**Fig. 1:**
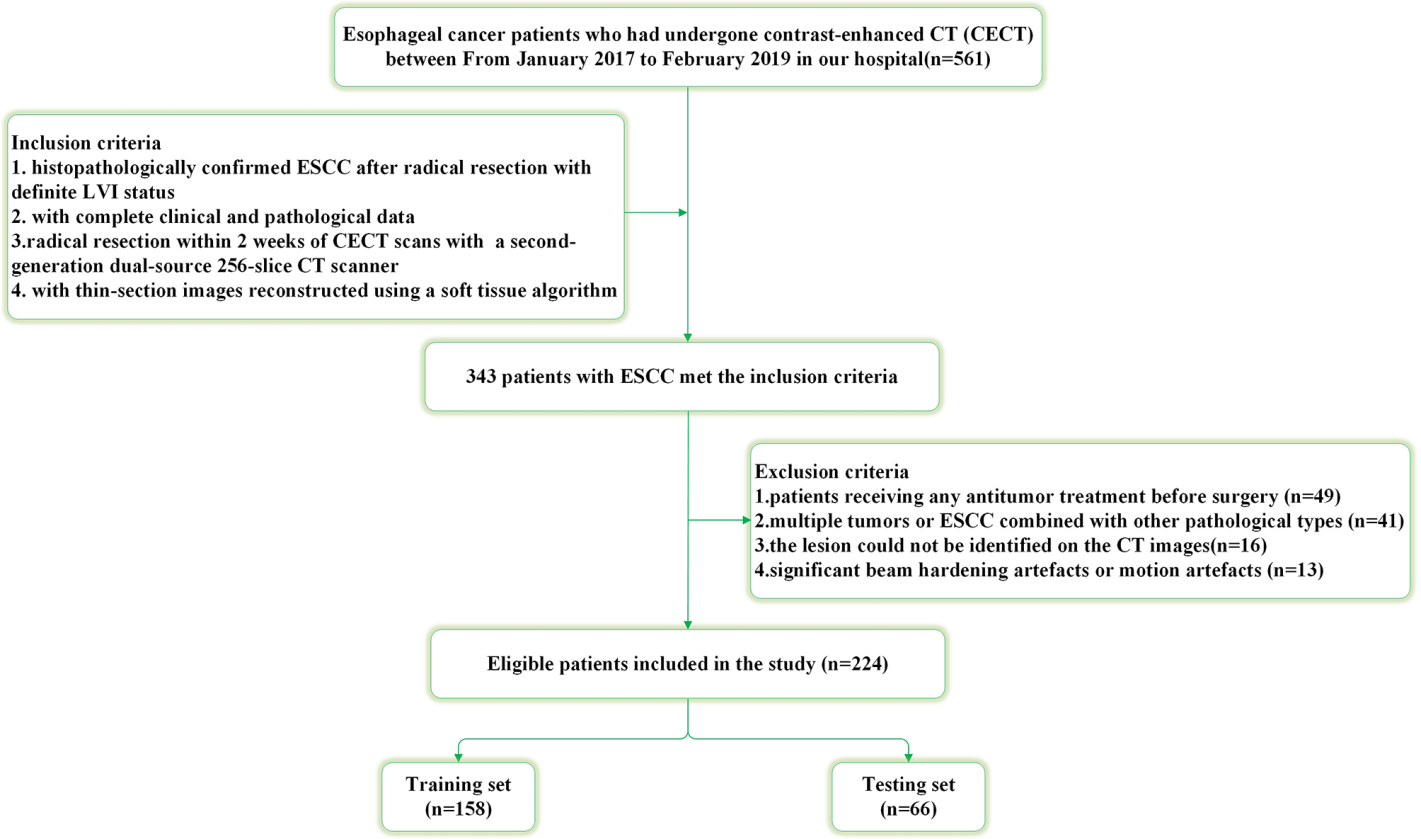
The flow chart of enrolled patients in our study

From January 2017 to February 2019, a total of 224 consecutive ESCC patients (157 males and 67 females, mean age 62.8 years) were enrolled based on the following inclusion criteria: ❶ histopathologically confirmed ESCC after radical resection with definite LVI status; ❷ with complete clinical and pathological data; ❸ radical resection within 2 weeks of CECT scans with a second-generation dual-source CT scanner; ❹ with thin-section images reconstructed using a soft tissue algorithm. The following criteria were exclusions: ❶ patients received any antitumor treatment before surgery (*n* = 49); ❷ multiple tumors or ESCC combined with other pathological types(*n* = 41); ❸ the lesion could not be identified on the CT images (*n* = 16); ❹ significant beam hardening artefacts or motion artefacts (*n* = 13). The enrolled ESCC patients were randomly allocated into a training set (158 patients) and a testing set (66 patients) in a 7:3 ratio. Figure 1 illustrates the flowchart for the patient selection process.

### Clinical and pathological features analysis

All ESCC patients underwent radical esophagectomy with lymphadenectomy within 2 weeks after the CT scan. The clinical and pathological features included patients’ gender, age, tumor location, pathological maximum tumor thickness (pThick), pathological tumor length (pLength), tumor-differentiation degree, peripheral nerve invasion (PNI), and LVI status, pathological T stage (pT), pathological N stage (pN), pathological TNM stage (pTNM). All pathological features were analyzed by 2 pathologists (with 9 and 11 years of experience in EC diagnosis). The pTNM stage was reclassified according to the 8th Ed. of the AJCC/UICC ESCC staging system.

### CT image acquisition

All patients in the study underwent chest CECT scans using a second-generation dual-source CT scanner (Somatom Definition Flash, Siemens, Forchheim, Germany). The scan protocol comprised the following parameters: tube voltage of 120 kVp, automatic mA, a matrix size of 512 × 512, a collimation of 128 × 0.6 mm a pitch of 1.2, a gantry rotation time of 0.5 s, a slice thickness of 5.0 mm, and a reconstructed slice thickness of 1.0 mm using a soft-tissue algorithm (b30f). The scan range extended from the thoracic inlet level to the abdominal trunk artery. Following a 30-second delay, an injection of contrast medium (1.5 ml/kg, 300 mg I/ml, Iohexol) at a rate of 3.0–4.0 ml/sec was administered via a high-pressure syringe pump into the elbow vein to acquire arterial phase CT images.

### Tumor segmentation

All tumors were segmented using the open-source software 3D-slicer (V. 5.2.2) on the 1.0 mm thickness arterial phase CECT images. The 3D regions of interest (ROIs) were segmented based on the whole-volume tumor, while the 2D ROIs were delineated using the largest tumor cross-section. The criteria for lesions were defined as esophageal wall thickness > 5 mm or esophageal diameter > 10 mm (without gas) with local irregular luminal narrowing [21–23]. The ROIs of the tumor included the intratumoral necrotic area. The air, fluid, surrounding fatty tissue, lymph nodes, heart and lung tissue, blood vessels, and bone tissue in the esophageal lumen were excluded.

The inter-/intra-class correlation coefficient (ICC) analysis was estimated to assess inter- /intra-reader reproducibility and reliability of radiomics feature extraction. Thirty patients were randomly selected from the entire cohort, and Radiologist 1 and Radiologist 2, each with 12 years of experience in diagnosing esophageal cancer, independently performed 2D and 3D ROI segmentation for the 30 patients. Segmentation of 2D and 3D ROIs for the remaining 194 cases was done independently by Radiologists 1. Radiologist 1 segmented the tumors of the same 30 patients once more after two weeks. The ICCs between Radiologist 1 and Radiologist 2, and within Radiologist 1 was evaluated. For radiomics features, ICCs > 0.75 imply good agreement and reproduction.

### Radiomics feature extraction and model construction

We extracted the 2D radiomics features and 3D radiomics features from 2D and 3D ROIs, respectively. The features were extracted using the Pyradiomics toolkit [24]. The CT images were first standardized using image isotropic resampling (1 × 1 × 1mm^3^) and grayscale discretization with binwith 25 [25].

Radiomics features include four types: shape-based features, first-order features, texture features, and high-level features. The texture features consisted of 24 Gy level co-occurrence matrix (GLCM) features, 14 Gy level dependence matrix (GLDM) features, 16 Gy level run length matrix (GLRLM) features, 16 Gy level size zone matrix (GLSZM) features, and 5 neighboring gray tone difference matrix (NGTDM) features. Eight distinct combinations of frequency bands were utilized to generate Wavelet-based features (LLL, LLH, LHL, LHH, HHH, HHL, HLH, HLL). The were 18 classes of first-order histogram features. The first-order histogram and texture may shift due to the transformations introduced by the Laplacian of Gaussian (LoG) and Wavelet filters applied to the features. Therefore, features transformed by LoG and Wavelet filters may be more comprehensive and effective in diagnosis. Additionally, the 2D radiomics features include 9 shape features, and the 3D features include 14 shape features. 1130 radiomics features were generated from the 3D ROIs and 753 radiomics features from the 2D ROIs to estimate tumor heterogeneity. To assess the relationship between 2D and 3D feature clusters, we utilized unsupervised clustering and a radiomics heatmap.

**Fig. 2:**
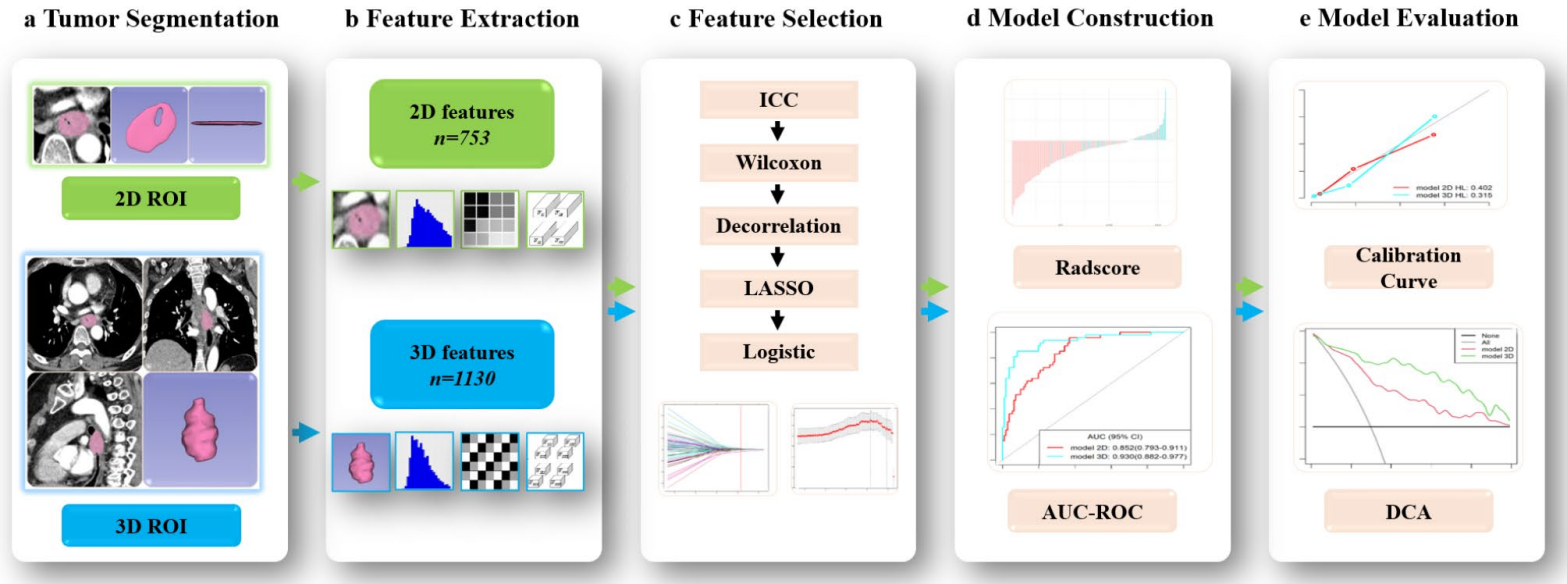
2D and 3D radiomics prediction pipeline for LVI. **a** The 3D ROIs were segmented based on the whole-volume tumor, while the 2D ROIs were delineated using the largest tumor cross-section. **b** Radiomics features include four types: shape-based features, first-order features, texture features, and high-level features. 1130 radiomics features were generated from the 3D ROIs and 753 radiomics features from the 2D ROIs. **c** To select the optimal radiomics features, we used ICC, Wilcoxon rank sum test, Spearman correlation analysis, LASSO, and multivariate stepwise logistic regression analysis to determine the feature set with minimum Akaike’s Information Criterion. **d** The Radscores for both 2D and 3D radiomics models were developed to represent the predictive status of each patient. The area under the curve (AUC) of the receiver operating characteristic (ROC) curve was used to quantify the prediction performance. **e** Calibration curves were generated to assess the goodness-of-fit of the two radiomics models. Decision curve analysis (DCA) was then conducted to evaluate the clinical utility of the two models

To select the optimal radiomics features, we employed a four-step approach as outlined below: ❶ The reliability of feature extraction was assessed using ICC analysis. Features with ICCs > 0.75 were retained. ❷ The Wilcoxon rank sum test identified features with significant differences between LVI-present and LVI-absent cases. ❸ Spearman correlation analysis eliminated features with ICCs > 0.9, reducing redundancy. ❹ The least absolute shrinkage and selection operator (LASSO) algorithm with minimum criteria and 10-fold cross-validation were used to determine the tuning parameter selection (λ) and optimal features. Finally, the remaining features were submitted to multivariable stepwise logistic regression analysis, and the feature set with the smallest Akaike’s Information Criterion (AIC) was retained as the best model. Figure 2 shows a flowchart of the proposed analysis radiomics analysis workflow mentioned previously.

### Statistics analysis

Patients were classified into LVI-present and LVI-absent groups. Mean ± SD was used to represent continuously distributed data, median (range) was used to describe non-normally distributed continuous variables, and frequencies (%) were used to express categorically dispersed variables for clinical and pathological characteristics. The Mann–Whitney U test was employed for nonnormal continuous variables and the independent sample t-test for normal ones. The categorical data were analyzed using the Chi-squared test.

The diagnostic performance was evaluated using receiver operating characteristic (ROC) curves and the area under the curve (AUC). The accuracy, sensitivity, specificity, positive predictive value (PPV), and the negative predictive value (NPV) were calculated. The AUC of the two radiomics models was compared using the Delong test. Calibration curves were generated to evaluate the goodness-of-fit of the two radiomics models. The reliability of the calibration curves was assessed using the Hosmer-Lemeshow (HL) test. The actual clinical utility of the two radiomics models was evaluated by decision curve analysis (DCA). All statistical analyses were conducted using R statistical software (Version 4.2.1). A two-tailed test with a significant level of *P* < 0.05 was considered statistically significant.

## Results

### Clinical and pathological features of patients

Table 1 displays patients’ clinical and pathological characteristics in the training and testing sets. Our study comprised 224 patients, including 158 (70.5%) LVI-absent and 66 (29.5%) LVI-present patients. There were no significant differences in age, sex, and location between the two groups. Pathological features, including pLength, pThick, pT stage, tumor differentiation, PNI, pN stage, and pAJCC stage, significantly differed between the two groups.

**Table 1:** Clinical and pathological features of the patients in the training and testing sets

Variable	LVI-absent (*N* = 158)	LVI-present (*N* = 66)	*P*
Gender, n			0.175^#^
male	106 (67.1%)	51 (77.27)	
female	52 (32.9%)	15 (22.73)	
Age (years), median (quartile)	65.00 [58.00;69.00]	62.000 [56.00;67.75]	0.140^#^
Location			0.420^#^
upper	10 (6.3%)	5 (7.6%)	
middle	114 (72.2%)	42 (63.6%)	
lower	34 (21.5%)	19 (28.8%)	
pLength (cm), median (quartile)	3.50 [3.00;4.00]	4.00 [3.00;5.00]	0.002^*^
pThick (cm), median (quartile)	1.000 [0.80;1.38]	1.10 [1.00;1.68]	0.005^*^
Differentiation, n			0.003^&^
I	2 (1.3%)	0	
II	112 (70.9%)	33 (50.0%)	
III	44 (27.6%)	33 (50.0%)	
PNI, n			0.172 ^#^
absent	110 (69.6%)	39 (59.1%)	
present	48 (30.4%)	27 (40.9%)	
pT stage, n			0.019^**#**^
1	12 (7.6%)	1 (1.5%)	
2	41 (26.0%)	9 (13.6%)	
3	104 (65.8%)	56 (84.8%)	
4	1 (0.6%)	0	
pN stage, n			<0.001^**#**^
0	100 (63.3%)	14 (21.2%)	
1	40 (25.3%)	23 (34.9%)	
2	12 (7.6%)	16 (24.2%)	
3	6 (3.8%)	13 (19.7%)	
pTNM stage, n			<0.001^**#**^
I	8 (5.1%)	0	
II	94 (59.5%)	14 (21.2%)	
III	50 (31.6%)	39 (59.1%)	
IV	6 (3.8%)	13 (19.7%)	

^#^, Chi-square test; ^*^, Mann–Whitney U test; PNI, peripheral nerve invasion; pT, pathological T stage; pN, pathological N stage; pTNM, pathological TNM stage

### Radiomics feature extraction and model construction

**Fig. 3:**
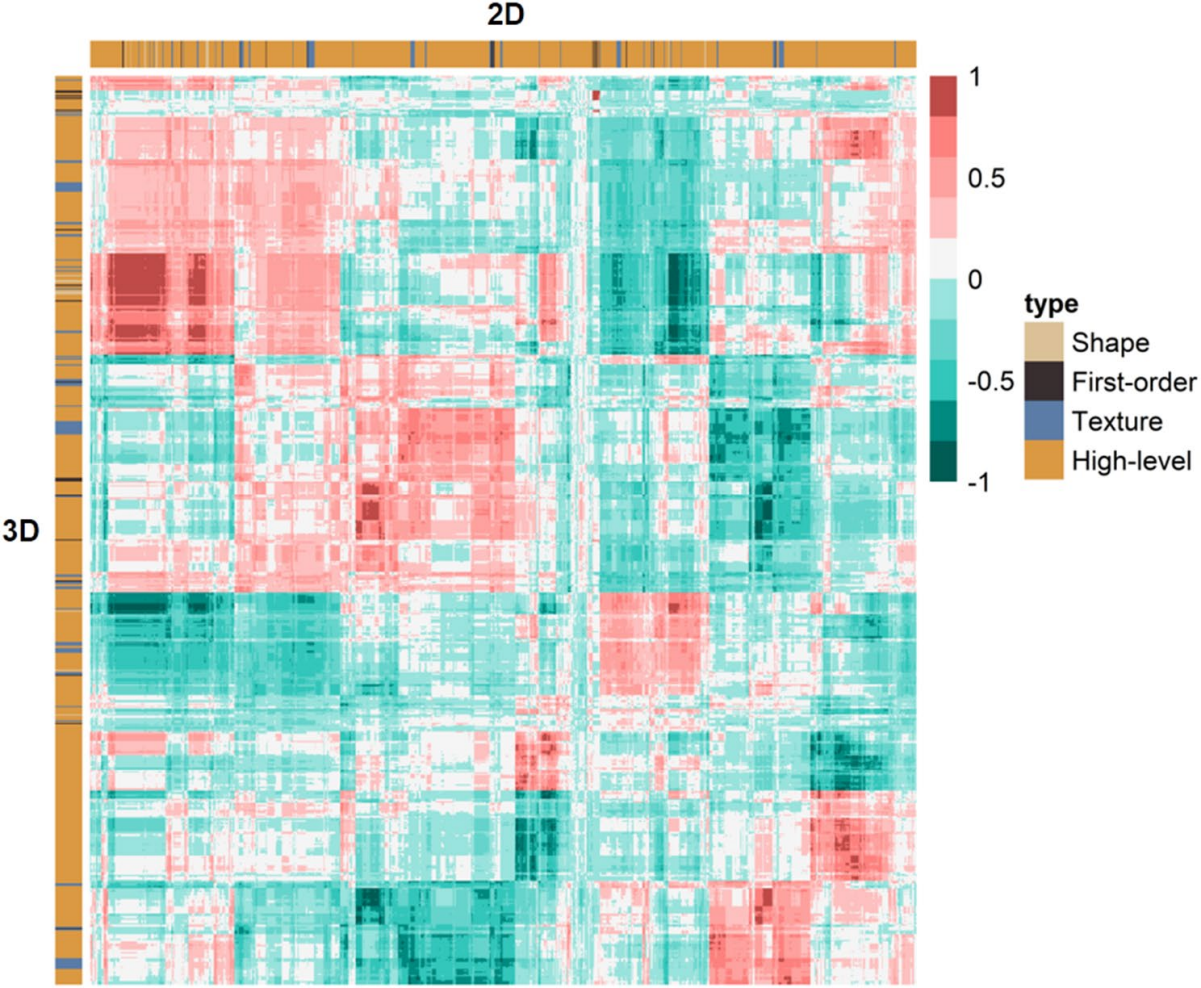
Heat map of 2D and 3D radiomics feature clusters containing four classifications. The redder areas indicate that the corresponding 2D and 3D radiomics features are more strongly correlated, while the darker green areas represent the contrary

A total of 753 2D and 1130 3D radiomics features were extracted from 2D and 3D ROIs, respectively. We investigated the relationship between the 2D and 3D radiomics features and visualized it via a heatmap shown in Fig. 3. 392 of 753 radiomics characteristics derived from 2D ROIs exhibited good agreement and reproduction with ICCs greater than 0.75. 937 of 1130 radiomics features derived from 3D ROIs exhibited good agreement and reproduction with ICCs greater than 0.75. According to the Wilcoxon rank-sum test, 258 2D radiomics features and 525 3D radiomics features showed a statistically significant difference between LVI-present and LVI-absent patients. These radiomics features were included in the following Spearman correlation analysis. Through Spearman correlation analysis, radiomics features with ICCs larger than 0.9 were discarded, leaving 56 2D radiomics features and 77 3D radiomics features for the subsequent LASSO analysis.

**Fig. 4:**
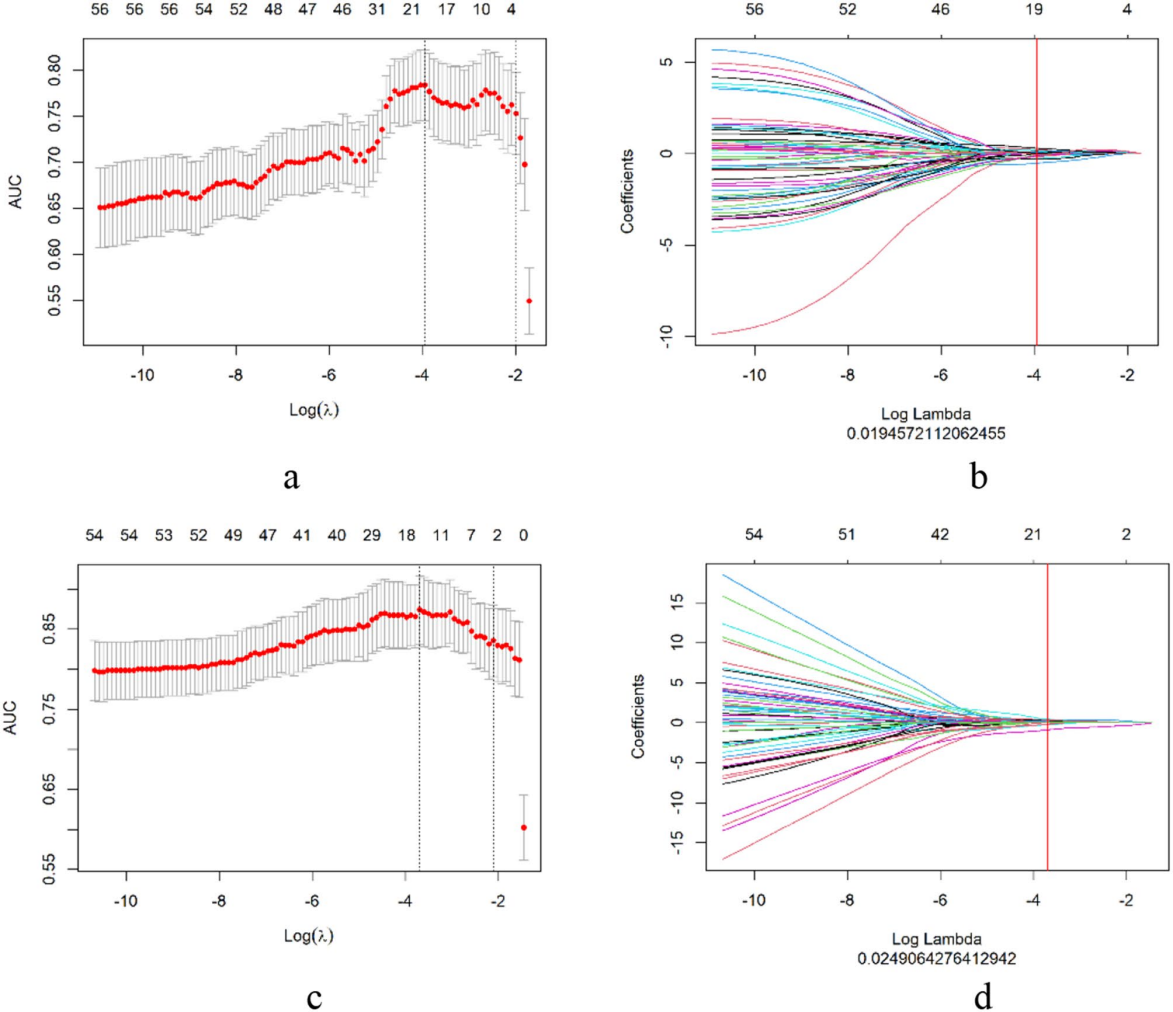
2D and 3D radiomics feature selection using LASSO logistic regression. The AUC curve was plotted versus log (λ). 2D log (λ) = − 1.721, with λ = 0.019 was chosen; 3D log (λ) = − 1.602, with λ = 0.025 was chosen, respectively (**a**,** c**). 2D and 3D radiomics LASSO coefficients profile of the 56 and 77 radiomics features, respectively (**b**,** d**). The vertical red line was drawn at the value selected using 10-fold cross-validation, where the optimal λ yield 19 and 15 features with non-zero coefficients, respectively

**Fig. 5:**
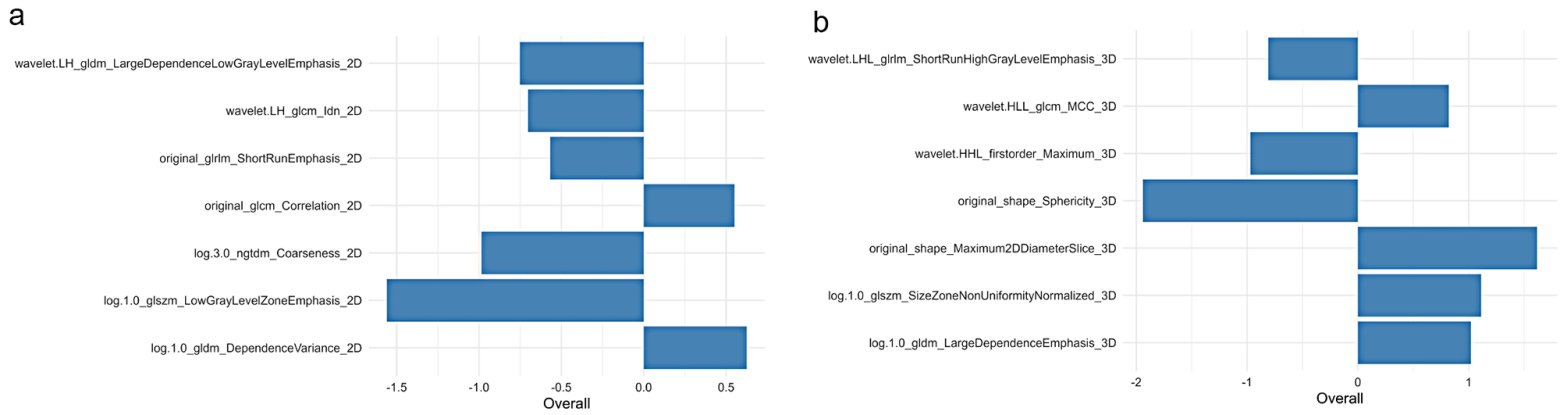
Construction of 2D and 3D radiomics models. The histograms show the contribution of the selected 2D (**a**) and 3D (**b**) radiomics features and their regression coefficients in the 2D and 3D radiomics models, respectively

For 2D radiomics features and 3D radiomics features, the optimal λ values of 0.019 and 0.025, with log (λ) =-1.721 and − 1.602, respectively, were chosen (Fig. 4). Lastly, the 2D and 3D radiomics models were built using multivariable stepwise logistic regression, retaining the feature set with the lowest AIC value. Seven radiomics features were retained in both the 2D and 3D radiomics models, respectively. Figure 5 depicts the selected 2D and 3D radiomics features and their coefficients. The particular interpretations and formulas for 2D and 3D radiomics features can be found in the Table S1 and Table S2, respectively. For each patient, the radiomics score (Radscore) was derived from a linear combination of selected features with their corresponding coefficients, which can be expressed as follows:

**2D-Radscore**=-1.683 + 0.551*original_glcm_Correlation_2D-0.568*original_glrlm_ShortRunEmphasis_2D-0.160*log.1.0_glszm_LowGrayLevelZoneEmphasis_2D + 0.625*log1.0_gldm_DependenceVariance-0.985*log.3.0_ngtdm_Coarseness_2D-0.702*Wavelet.LH_glcm_Idn_2D-0.725*Wavelet.LH_gldm_LargeDependenceLowGrayLevelEmphasis_2D;

**3D-Radscore**=-1.906 + 1.617*original_shape_Max2DDiameterSlice_3D-1.940*original_shape_Sphericity_3D.

+ 1.113*log.1.0_glszm_SizeZoneNonUniformityNormalized_3D-1.021* log. l. 0_gldm_LargeDependenceEmphasis_3D-0.810*Wavelet.LHL_glrlm_ShortRunHighGrayLevelEmphasis_3D + 0.828*Wavelet.HLL_glcm_MCC_3D-0.971*Wavelet. HHL_firstorder_Maximum_3D.

**Fig. 6:**
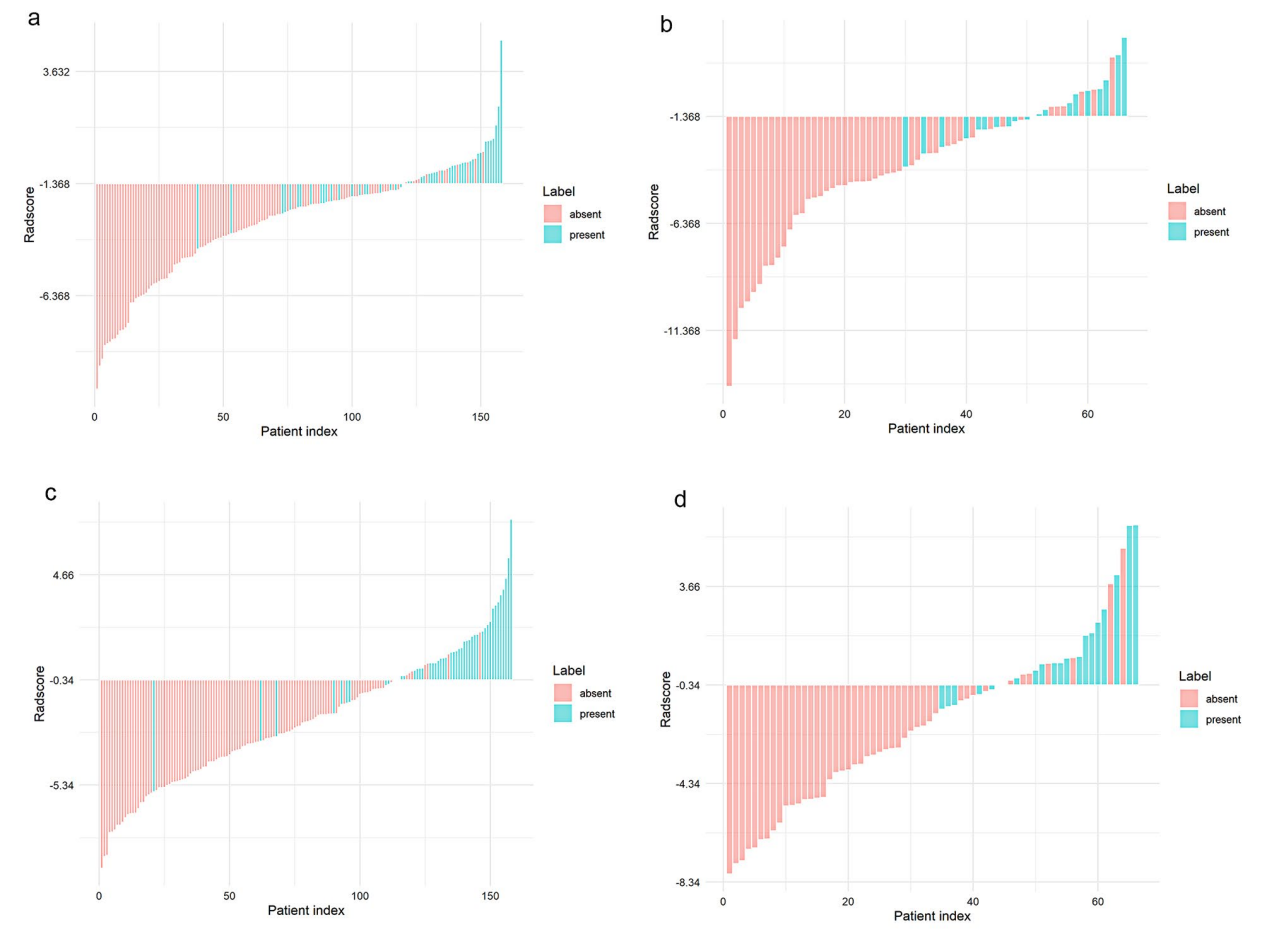
Bar charts of Radscore for each patient of 2D and 3D radiomics models in the training (**a**,** b**) and testing sets (**c**,** d**). The light red bars represent the Radscore of LVI-absent ESCC patients, whereas the light blue bars indicate the Radscore of LVI-present ESCC patients. The light red bars below the threshold represent correctly identified LVI-absent ESCC patients, whereas those above represent incorrect identifications. The light blue bars above the threshold are correctly identified LVI-present ESCC patients, whereas those below are incorrect identifications

The 2D and 3D Radscores for each patient between training and testing sets are displayed in Fig. 6.

### Model evaluation

**Fig. 7:**
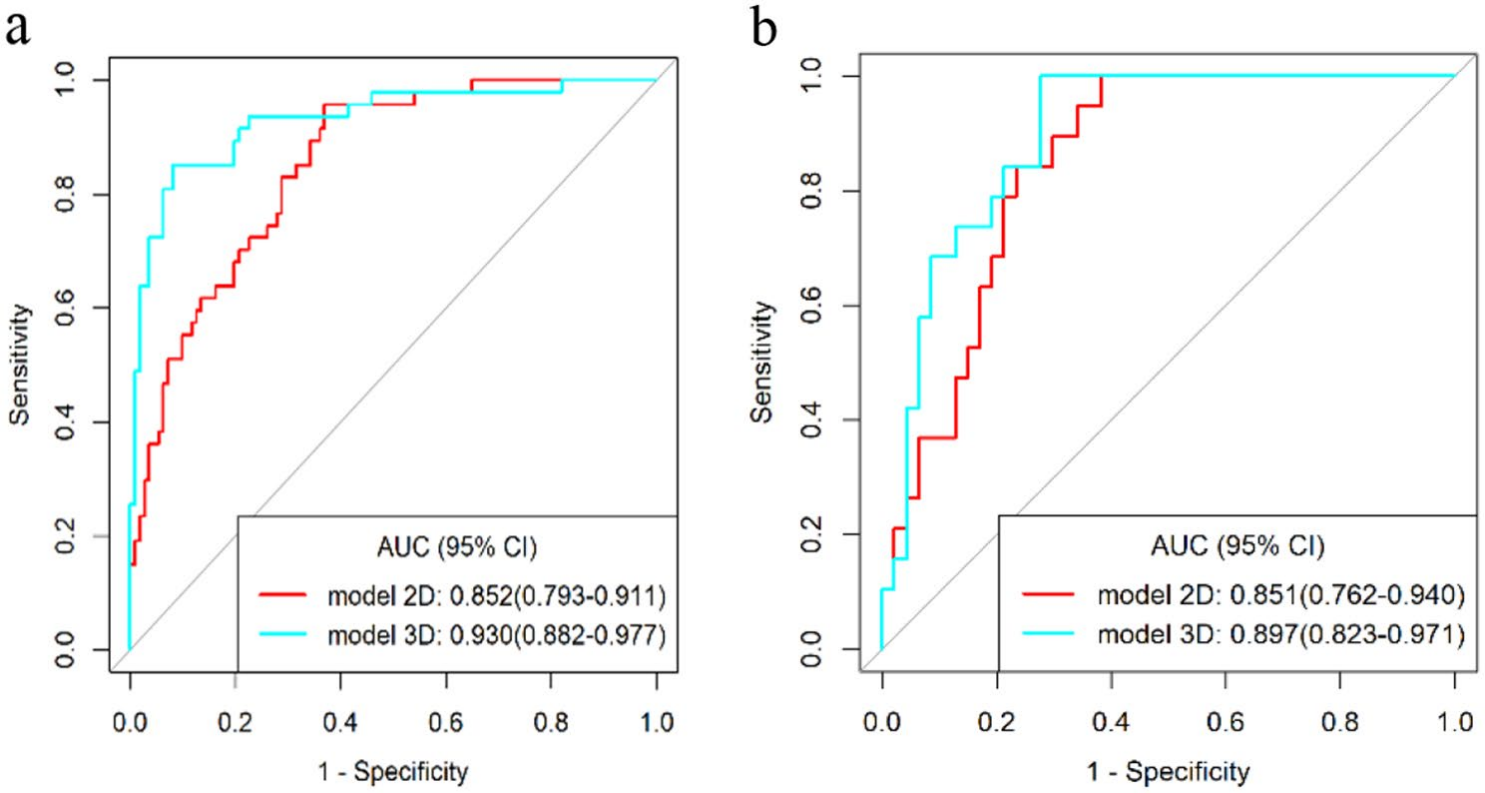
The ROC curves of the 2D and 3D radiomics models in the training (**a**) and testing (**b**) sets. The 3D radiomics model (blue curve) provides higher AUC values than the 2D radiomics model (red curve). The AUC values and 95% confidence interval (CI) values are shown in the lower right-hand corner of the figure

Figure 7 displays the ROC curves of the two models. Based on the Delong test, the 3D radiomics model showed significantly greater AUCs values than the 2D radiomics model (3D, 0.930; 2D, 0.852, *P* = 0.006) in the training set. The difference in the AUC values between the two radiomics models was not significant in the testing set (3D, 0.897; 2D, 0.851, *P* = 0.343). In both the training and testing sets, the accuracy of the 3D radiomics model was 0.899 and 0.788, respectively, both surpassing the accuracy of the 2D model (0.728 and 0.758). In both the training and testing sets, the 2D radiomics model showed greater sensitivity and NPV than the 3D radiomics model. Conversely, the 3D radiomics model revealed higher specificity and PPV than the 2D radiomics model. Table 2 displays the detailed diagnostic performance of the 2D and 3D radiomics models.

**Table 2:** Diagnostic performance of the 2D and 3D radiomics models

Model	AUC	Sensitivity	Specificity	Accuracy	PPV	NPV
Training						
2D	0.852(0.793–0.911)	0.957(0.787-1.000)	0.631(0.333–0.721)	0.728(0.651–0.796)	0.523(0.474–0.534)	0.972(0.949–0.976)
3D	0.930(0.882–0.977)	0.851(0.638–0.936)	0.919(0.721–0.973)	0.899(0.841–0.941)	0.816(0.769–0.830)	0.936(0.920–0.939)
Testing						
2D	0.851(0.762–0.940)	0.842(0.576-1.000)	0.723(0.574–0.872)	0.758(0.636–0.855)	0.552(0.457–0.594)	0.919(0.900-0.932)
3D	0.897(0.823–0.971)	0.789(0.579-1.000)	0.787(0.660–0.957)	0.788(0.670–0.879)	0.600(0.524–0.655)	0.902(0.886–0.918)

Values within parentheses are 95% confidence interval values; PPV, positive predictive value; NPV, negative predictive value

**Fig. 8:**
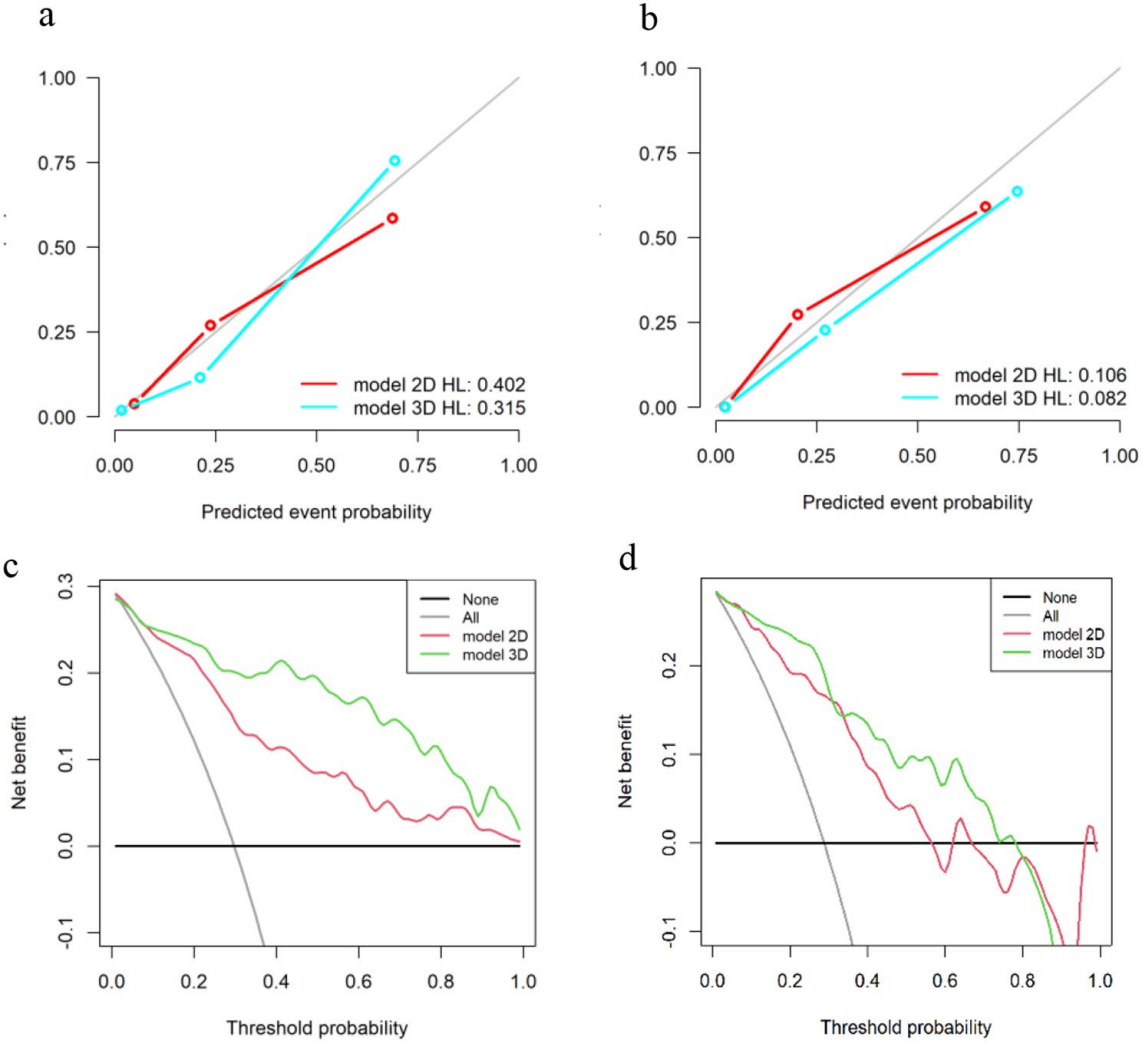
Calibration curves and DCA curves of the radiomics models. Calibration curves in the of the 2D and 3D radiomics models in the training (**a**) and testing (**b**) sets indicate that no significant departure from a perfect fit. The DCA showed that in the training (**c**) and testing (**d**) sets, the overall net benefit of the 3D radiomics model (green curve) was higher than that of the 2D radiomics model (red curve) in predicting LVI within most threshold probability ranges. It means that the 3D model has a high actual clinical utility. The y-axis represents the overall net benefit and the x-axis represents the threshold probability

The calibration curves for the training and testing sets of 2D and 3D radiomics models demonstrated good concordance (Fig. 8a, b).The HL test produced a nonsignificant P value, indicating no deviation from the exact fit (Fig. 8a, b). The DCA showed that the overall net benefit of the 3D radiomics model (green curve) was higher than that of the 2D radiomics model (red curve) in predicting LVI within most threshold probability ranges (Fig. 8c, d). It means that the 3D model has a high actual clinical utility.

## Discussion

LVI within tumors not only signifies an increased likelihood of metastases but also serves as an independent prognostic factor, significantly associated with higher risks of tumor recurrence and diminished overall survival [26–29]. From a clinical perspective, preoperatively predicting the LVI of patients with ESCC is essential to selecting an appropriate treatment strategy. In this study, based on preoperative contrast-enhanced CT images, we used 2D and 3D ROIs of tumors to extract radiomics features and build prediction models for predicting the LVI status of ESCC patients, respectively. The results revealed that the 2D and 3D radiomics models could effectively predict the preoperative LVI in ESCC patients, with AUCs of 0.852 versus 0.930 for the training set and 0.851 versus 0.897 for the testing set, respectively. DCA suggested that the 3D radiomics model is more clinically beneficial than the 2D radiomics model. The value of this study is to compare the performance of 2D and 3D models for the prediction of the LVI in ESCC and to provide guidance for the clinical selection of suitable prediction methods to optimize the treatment modality.

In recent years, radiomics has emerged as an important imaging technique for identifying histological and biological features of tumors [12]. Traditional imaging methods cannot directly observe or predict LVI status; however, many studies have found that radiomics features can effectively predict LVI status. Zhang et al. [14] found that multimodal (CT/MR) radiomics could effectively predict LVI status in rectal cancer and show great potential in improving treatment decisions. Li et al. [30] developed a whole-volume 3D CT radiomics model for LVI prediction in gastric cancer (GC) patients, resulting in AUCs of 0.698 and 0.676 in training and testing sets, respectively. In another study, Chen et al. [13] discovered that radiomics features derived from 3D ROIs had the potential to serve as valuable markers for predicting the LVI and progression-free survival in patients with GC. Li et al. [17] adopted 3D intratumoral and peritumoral radiomics features for LVI prediction in rectal cancer. The AUC values of their models were 0.761 in the training group and 0.748 and 0.763 in the internal and external validation, respectively. These studies confirmed the potential of the 3D radiomics model in predicting LVI in digestive tract tumors but did not reveal the value of the 2D radiomics model or its differences from the 3D radiomics model.

However, whether 3D or 2D ROIs are better for clinical use is still being determined [31]. Compared to 2D radiomics analysis, 3D radiomics analysis covers the entire tumor volume and provides a more comprehensive description of tumor heterogeneity. Huang et al. [32] found that the 3D radiomics model performed better than the best combined 2D model in predicting the invasiveness of pancreatic solid pseudopapillary neoplasm. Liu et al. [33] reported that the 3D radiomics analysis provided better stratification of the histologic grade of cervical cancer compared with the 2D radiomics analysis. Consistent with the above studies, our results suggest that 3D radiomics model performed better than 2D radiomics model in predicting LVI. The 3D radiomics model had higher AUCs (0.930 and 0.897), accuracy (0.899 and 0.788), specificity (0.919 and 0.787) and PPV (0.787 and 0.600) than that of the 2D radiomics model in the training and testing sets. However, the 2D radiomics model showed higher sensitivity and NPV. Therefore, it can be inferred that the esophagus being a hollow organ, ESCC may not exhibit LVI only at the level of the largest tumor. Consequently, relying solely on one-slice 2D radiomics features may not fully characterize the pathology of the entire tumor.

However, in other studies, 3D models did not perform better than 2D models. Zhu et al. [34] suggested that the 2D annotation was a more time-efficient and effective method for predicting immunotherapy and chemotherapy response in ESCC patients. Meng et al. [16] showed that 2D radiomics models revealed slightly higher AUCs than 3D radiomics models in predicting the LVI, lymph node metastasis and T-stage classification of GC. Piazzese et al. [35] reported that 2D radiomics features extracted from CT images of EC patients performed slightly better than 3D ones. These studies suggest that the 2D radiomics analysis may have better predictive value in digestive tract tumors. This may be because 3D annotation introduces additional noise that drowns out relevant information and affects the results. Moreover, annotating multiple slices may exacerbate this effect [16].

Theoretically, 3D radiomics features may be more reproducible than 2D radiomics features. In the present study, out of 753 2D features, 392 demonstrated good agreement and reproducibility, while among the 1130 3D features, 937 exhibited ICCs > 0.75. The prediction accuracy of the 3D radiomics model was slightly higher compared to that of the 2D radiomics model. The extraction and selection of radiomics features are essential procedures in radiomics research. In our previous study, we conducted a radiomics analysis using CECT images acquired from two CT scanners to predict the LVI status of ESCC [19]. Probably due to the differences in CT models and parameters, only two radiomics features were ultimately retained after filtering sequences. In the present study, we selected ESCC patients who underwent CT scans using only one scanner and scanning protocol, which improved the homogeneity of the CT images to some extent. In the previous study [19], the AUC values of the 3D imaging histology model were 0.847 and 0.826 in the training group. It is noteworthy that the AUC values of the 3D model in this study improved to 0.930 and 0.897, respectively. In addition, the accuracy, sensitivity, specificity, PPV, and NPV of the 3D model in this study were improved compared to the previous study.

The specific radiomics features included in the 2D and 3D radiomics models are presented in Fig. 5. However, the retained 2D and 3D radiomics features included in the two models were different. It may be due to a variety of factors. One possible explanation for this divergence is related to filters and filtration processes. Log-transform and wavelet-transform are often used in medical images before texture feature extraction, enabling the extraction of more valuable features [36, 37]. Another significant factor could be the varying extents and levels of tumor heterogeneity present in 2D and 3D Regions of Interest (ROIs), including variations in internal tumor density, cytoarchitecture, and vascular structure. Therefore, the two radiomics models preserved various radiomics features.

In the 3D radiomics model, 7 radiomics features were finally retained, including 3 wavelet-transform features, 2 original shape features, and 2 log-transform features. Two shape features were preserved in the 3D radiomics model: Sphericity and maximum 2D diameter slice. As seen in Fig. 5, among the features with a positive correlation, Max2DDiameterSlice had the greatest influence. In contrast, Sphericity had the greatest impact among the features with a negative correlation. Additionally, three positively correlated features and two negatively correlated features were included. The Maximum2DDiameter slice refers to the largest pairwise Euclidean distance in the row-column (usually axial) plane between tumor surface grid vertices. The larger the Maximum2DDiameter, the greater the likelihood of LVI. Sphericity is a shape features that describes how closely a given volume resembles a perfect sphere [38]. ESCC tumors with lower Sphericity were more likely to develop LVI, which is consistent with previous findings [19]. In the previous study [19], only two features, Sphericity and GLNU, were retained in the 3D model. The performance of the 3D model in the present study has improved compared to the previous 3D model. This difference may be attributed to the significant difference in the data from using two different CT scanners while acquiring the previous CT dataset.

In the 2D radiomics model, 7 radiomics features were finally retained, including 2 wavelet-transform features, 1 original GLRLM feature, 1 original GLCM feature, and 2 log-transform features. There are 5 negatively correlated features and 2 positively correlated features. The most influential radiomics features were the two negatively correlated features log-transform: LowGrayLevelZoneEmphasis (log.1.0) and Coarseness (log.3.0). LowGrayLevelZoneEmphasis measures the distribution of lower gray-level size zones, with a higher value indicating a greater proportion of lower gray-level values and size zones in the image. As the value of LowGrayLevelZoneEmphasis increases, the likelihood of LVI. Coarseness is a measure of the average difference between the center voxel and its neighbourhood and is an indication of the spatial rate of change. A higher value indicates a lower spatial change rate and a locally more uniform texture. It means that the smaller the Coarseness value, the greater the likelihood of LVI. Among the positively correlated radiomics features, DependenceVariance (log1.0) was the most influential. The GLDM measures gray-level dependence, while the DependenceVariance measures dependence variance, with a higher value indicating greater dependence difference and heterogeneous texture in local zone size [39, 40]. It can be hypothesized that the greater the DependenceVariance, the higher the likelihood of LVI in ESCC. However, in the 2D radiomics model, no valuable shape features were retained. As shown in Fig. 7 and Table 2, the 3D radiomics model demonstrated superior performance in predicting the LVI status of ESCC. One potential reason for the superior performance of 3D models compared to 2D models may be attributed to the incorporation of meaningful shape features.

In a previous study [19], we found that the diagnostic performance of the radiomics model generated with the (LR) method was equivalent to that of a support vector machine (SVM) and higher than that of a decision tree (DT) in predicting the LVI of ESCC. Due to its straightforward implementation and interpretability, LR is routinely used in clinical settings. Consequently, we adopted LR for the present study, prioritizing its ease of integration into clinical workflows while maintaining robust predictive accuracy for LVI in ESCC.

Radiomics has an interpretability advantage over deep learning, but it typically requires medical professionals to select and extract features manually [41]. Deep learning autonomously learns task-specific features, reducing reliance on domain expertise [41]. Studies have shown that deep learning can effectively detect esophageal cancer on chest CT scans, minimizing missed diagnoses [42, 43]. In predicting tumor LVI, radiomics and deep learning perform similarly [44, 45], and their combination enhanced the performance [45]. Typically, radiomics features are generated from the ROIs of the lesions, which require significant expertise and manual annotation [46]. Manual annotation is the primary modality for radiomics analysis of ESCC. Throughout all the processes of radiomics analysis, the longest time was spent outlining the extent of the tumor ROIs. The manual process of drawing 3D ROIs is significantly more time-consuming compared to drawing 2D ROIs. The present study did not compare the number of layers and the time spent outlining the tumors in the two models. However, as shown in Table 1, the median pathologic lengths of the tumors were 3.5 cm and 4.0 cm for patients with LVI and without LVI, respectively. The number of layers in the 3D radiomics model was 35 and 40 times greater than that in the 2D radiomics model, based on a thickness of 1.0 mm.

This study has several limitations. Firstly, this was a single-center retrospective analysis involving only patients who underwent surgery; patients with invisible lesions were not included. This may introduce selection bias into the data, making the results less comparable. In the future, multicenter prospective studies incorporating larger sample sizes are worthwhile. Secondly, the sampling error resulted in a higher prediction performance for the training set compared to the testing set. Thirdly, the radiomics features extracted in this study were derived from arterial-phase CECT images, while the plain CT and venous-phase CECT images were not analyzed. The performance of radiomics models based on multiphase CECT may be enhanced. Fourthly, this study did not include traditional CT features and other clinical outcomes, which could improve the predictive performance of the model. In the future, our subsequent research will focus on exploring whether integrating time-saving 2D radiomics with conventional CT features can improve the model’s performance.

## Conclusion

In conclusion, 2D and 3D radiomics features emerge as promising predictors for LVI, with the 3D radiomics model demonstrating superior performance compared to its 2D counterpart. However, given the single-center retrospective nature of this study, validation in a prospective multi-center study is essential to enhance the reproducibility and broader applicability of the developed models. Whether the 2D radiomics model combined with conventional CT features can improve the prediction performance makes our next research possible direction.

## Electronic supplementary material

Below is the link to the electronic supplementary material.

**Supplementary Material Table S1**: Radiomics features preserved by 2D model and their interpretations and formulas.

**Supplementary Material Table S2**: Radiomics features preserved by 3D model and their interpretations and formulas.

## Data Availability

The datasets generated during and/or analyzed in the current study are available from the corresponding authors upon reasonable request.

## Abbreviations

2D: Two-dimensional
3D: Three-dimensional
AIC: Akaike’s information criterion
AUC: Area under the curve
CECT: Contrast-enhanced CT
DCA: The decision curve analysis
ESCC: Esophageal squamous cell carcinoma
HL: Hosmer-Lemeshow
ICC: Inter-/intra-class correlation coefficient
LASSO: Least absolute shrinkage and selection operator algorithm
LVI: Lymphovascular invasion
NPV: Negative predictive value
pLength: Pathological tumor length
pN stage: Pathological N stage
pT stage: Pathological T stage
pThick: Pathological tumor maximum thickness
pTNM stage: Pathological TNM stage
PNI: Peripheral nerve invasion
PPV: Positive predictive value
ROC: Receiver operating characteristic curve
ROIs: Regions of interest
