# Supplementary material for: Prediction of lymphovascular invasion in esophageal squamous cell carcinoma by computed tomography-based radiomics analysis: 2D or 3D ?
Source: Cancer Imaging. 2024 Oct 17;24:141. doi: 10.1186/s40644-024-00786-5 (PMC11488362; doi:10.1186/s40644-024-00786-5)
Supplement: Supplementary file 1 — Supplementary Material Table S1: Radiomics features preserved by 2D model and their interpretations and formulas. [file 40644_2024_786_MOESM1_ESM.docx]

**Prediction of lymphovascular invasion in esophageal squamous cell carcinoma by computed tomography-based radiomics analysis：2D or 3D ?**

**Table S1** Radiomics features preserved by 2D model and their interpretations and formulas

|  | **Features** | **Features (Abbreviation)** | **Interpretation** | **Formula** |
| --- | --- | --- | --- | --- |
| 1 | original_glcm_Correlation | Correlation | Correlation is a value between 0 (uncorrelated) and 1 (perfectly correlated) showing the linear dependency of gray level values to their respective voxels in the GLCM. | $\frac{\sum_{i=1}^{N_{g}} \sum_{j=1}^{N_{g}} p(i,j)ij-\mu_{x}\mu_{y}}{\sigma_{x}(i)\sigma_{y}(j)}$ |
| 2 | original_glrlm_ShortRunEmphasis | ShortRunEmphasis (SRE) | SRE is a measure of the distribution of short run lengths, with a greater value indicative of shorter run lengths and more fine textural textures. | $\frac{\sum_{i=1}^{N_{g}} \sum_{j=1}^{N_{r}} \frac{\mathbf{P}(i,j\vert\theta)}{j^{2}}}{N_{r}(\theta)}$ |
| 3 | log.1.0_glszm_LowGrayLevelZoneEmphasis | LowGrayLevelZoneEmphasis (LGLZE) | LGLZE measures the distribution of lower gray-level size zones, with a higher value indicating a greater proportion of lower gray-level values and size zones in the image. | $\frac{\sum_{i=1}^{N_{g}} \sum_{j=1}^{N_{s}} \frac{\mathbf{P}(i,j)}{i^{2}}}{N_{z}}$ |
| 4 | log1.0_gldm_DependenceVariance | DependenceVariance (DV) | Measures the variance in dependence size in the image. | $\sum_{i=1}^{N_{g}} \sum_{j=1}^{N_{d}} p(i,j)(j-\mu)^{2},$  $\mathrm{where}\mu=\sum_{i=1}^{N_{g}} \sum_{j=1}^{N_{d}} jp(i,j)$ |
| 5 | log.3.0_ngtdm_Coarseness | Coarseness | Coarseness is a measure of average difference between the center voxel and its neighbourhood and is an indication of the spatial rate of change. A higher value indicates a lower spatial change rate and a locally more uniform texture. | $\frac{1}{\sum_{i=1}^{Ng} p_{i}s_{i}}$ |
| 6 | Wavelet.LH_glcm_Idn_2D | InverseDifferenceNormalized (IDN) | IDN (inverse difference normalized) is another measure of the local homogeneity of an image. Unlike Homogeneity1, IDN normalizes the difference between the neighboring intensity values by dividing over the total number of discrete intensity values. | $\sum_{k=0}^{Ng-1} \frac{p_{x-y}(k)}{1+\left( \frac{k}{N_{g}} \right)}$ |
| 7 | Wavelet.LH_gldm_LargeDependenceLowGrayLevelEmphasis | LargeDependenceLowGrayLevelEmphasis | Measures the variance in dependence size in the image. | $=\frac{\sum_{i=1}^{N_{g}} \sum_{j=1}^{N_{d}} \frac{\mathbf{P}(i,j)j^{2}}{i^{2}}}{N_{z}}$ |
