# Supplementary material for: Prediction of lymphovascular invasion in esophageal squamous cell carcinoma by computed tomography-based radiomics analysis: 2D or 3D ?
Source: Cancer Imaging. 2024 Oct 17;24:141. doi: 10.1186/s40644-024-00786-5 (PMC11488362; doi:10.1186/s40644-024-00786-5)
Supplement: Supplementary file 2 — Supplementary Material Table S2: Radiomics features preserved by 3D model and their interpretations and formulas. [file 40644_2024_786_MOESM2_ESM.docx]

**Prediction of lymphovascular invasion in esophageal squamous cell carcinoma by computed tomography-based radiomics analysis：2D or 3D ?**

**Table S2** Radiomics features preserved by 3D model and their interpretations and formulas

|  | **Features** | **Features (Abbreviation)** | **Interpretation** | **Formula** |
| --- | --- | --- | --- | --- |
| 1 | original_shape_Max2DDiameterSlice_3D | Maximum2DDiameterSlice | Maximum2DDiameterSlice is defined as the largest pairwise Euclidean distance between tumor surface mesh vertices in the row-column (generally the axial) plane. | **/** |
| 2 | original_shape_Sphericity_3D | Sphericity | Sphericity is the ratio of the perimeter of the tumor region to the perimeter of a circle with the same surface area as the tumor region and therefore a measure of the roundness of the shape of the tumor region relative to a circle. It is a dimensionless measure, independent of scale and orientation. The value range is 0<sphericity≤1, where a value of 1 indicates a perfect circle (a circle has the smallest possible perimeter for a given surface area, compared to other shapes). | $\frac{2\pi R}{P}=\frac{2\sqrt{\pi A}}{P}$ |
| 3 | log.1.0_glszm_SizeZoneNonUniformityNormalized_3D | SizeZoneNonUniformityNormalized (SZNN) | SZNN measures the variability of size zone volumes throughout the image, with a lower value indicating more homogeneity among zone size volumes in the image. This is the normalized version of the SZN formula. | $\frac{\sum_{j=1}^{N_{s}} \left( \sum_{i=1}^{N_{g}} \mathbf{P}(i,j) \right)^{2}}{N_{z}^{2}}$ |
| 4 | log. l. 0_gldm_LargeDependenceEmphasis_3D | LargeDependenceEmphasis (LDE) | A measure of the distribution of large dependencies, with a greater value indicative of larger dependence and more homogeneous textures. | $\frac{\sum_{i=1}^{N_{g}} \sum_{j=1}^{N_{d}} \mathbf{P}(i,j)j^{2}}{N_{z}}$ |
| 5 | Wavelet.LHL_glrlm_ShortRunHighGrayLevelEmphasis_3D | ShortRunHighGrayLevelEmphasis (SRHGLE) | SRHGLE measures the joint distribution of shorter run lengths with higher gray-level values. | $\frac{\sum_{i=1}^{N_{g}} \sum_{j=1}^{N_{r}} \frac{\mathbf{P}(i,j\vert\theta)i^{2}}{j^{2}}}{N_{r}(\theta)}$ |
| 6 | Wavelet.HLL_glcm_MCC | MaximalCorrelationCoefficient (MCC) | The Maximal Correlation Coefficient is a measure of complexity of the texture and 0≤MCC≤1. | $\begin{aligned} \sqrt{\text{second largest eigenvalue of Q}} \\ Q(i,j)=\sum_{k=0}^{N_{g}} \frac{p(i,k)p(j,k)}{p_{x}(i)p_{y}(k)} \end{aligned}$ |
| 7 | Wavelet. HHL_firstorder_Maximum_3D | Maximum | The maximum gray level intensity within the ROI. | $max(\mathbf{X})$ |
